# How does the position of the pelvis and femur influence the selection of prosthesis size during 2D preoperative planning for total hip arthroplasty?

**DOI:** 10.1186/s12891-024-07955-4

**Published:** 2024-10-24

**Authors:** Junzhe Wu, Chaohui Lin, Xunrong Zhuang, Lijiang He, Jiawei Wang, Xinzhe Zhou, Nanjie Xu, Huating Xie, Hanzhang Lv, Hui Ye, Rongmou Zhang

**Affiliations:** 1 Department of Orthopaedic Surgery, The Second Affiliated Hospital, Fujian Medical University, Quanzhou, 362000 Fujian China; 2 Department of Radiology, The Second Affiliated Hospital, Fujian Medical University, Quanzhou, 362000 Fujian China; 3 Department of Emergency surgery, The Second Affiliated Hospital, Fujian Medical University, Quanzhou, 362000 Fujian China; 4 The Second Clinical Medical College, Fujian Medical University, The Second Affiliated Hospital, Fujian Medical University, Quanzhou, Fujian China

**Keywords:** Total hip arthroplasty, Preoperative planning, Human phantom, Pelvis, Femur, mediCAD

## Abstract

**Purpose:**

Total Hip Arthroplasty (THA) is the primary treatment for hip diseases today. Nevertheless, total hip arthroplasty has its challenges, and one of these challenges is the potential for incorrect execution of the preoperative planning process. Such errors can lead to complications such as loosening and instability of the prosthesis and leg length discrepancy. In this study, we used human phantoms to investigate the influence of pelvic and femoral factors on prosthesis size selection in the preoperative planning of total hip arthroplasty and to provide a reference standard for clinical imaging in preoperative planning of total hip arthroplasty.

**Methods:**

In this experiment, we utilised a custom-made experimental device that enabled us to manipulate the movement of the pelvis and femur in various directions. The device also incorporated sensors to control the angle of movement. By obtaining X-rays from different positions and angles, we were able to determine the size of the prosthesis based on the 2D preoperative planning generated by the mediCAD software.

**Results:**

When the pelvis was in a nonneutral position, the size of the acetabular cup varied within a range of three sizes. Similarly, when the femur was in a nonneutral position, the size of the femoral stem varied within a range of two sizes. The movement of the pelvis and femur in the coronal plane, relative to the neutral position, did not impact the selection of the prosthesis size. However, the motion of the pelvis and femur in the sagittal and transverse planes had a notable effect.

**Conclusion:**

The selection of the prosthesis size for preoperative planning can be significantly influenced by specific positions of the pelvis and femur. It is crucial for the radiographer to ensure that the pelvis and femur maintain a standard neutral position, particularly in the sagittal and transverse planes, during the image acquisition process.

## Introduction

Total hip arthroplasty (THA) has been commonly performed worldwide with mature techniques and is currently the primary treatment for hip diseases. The leading causes include femoral neck fracture, osteoarthritis, femoral head necrosis and acetabular dysplasia. However, there are several problems associated with total hip arthroplasty, one of which lies in the incorrect implementation of preoperative planning [1]. Common failure modes of THA include wear, aseptic loosening, dislocation, infection, metal debris reaction, periprosthetic fracture, and unexplained hip joint pain [2]. A large-scale retrospective study [3] showed that the proportion of failure causes was aseptic loosening (23.19%), instability (22.43%), and infection (22.13%). Loosening and instability failures are frequently attributed to the sub-optimal or non-optimal positioning and sizing of the prosthesis. On the other hand, the causal relationship between incorrect implant size selection in preoperative template design and poorer clinical outcomes has not been clearly verified. Colombi et al. stated that there was no evidence that a complete match between the planned implant and the actual implant would have any positive impact on clinical outcomes [4]. Preoperative planning software programs are regarded as a crucial step in total hip arthroplasty to minimise complications, ensure surgical safety, and effectively prevent common failure modes [3].

Currently, alongside the conventional template technique for preoperative planning of total hip arthroplasty in clinical settings, numerous preoperative planning software has emerged with the integration of digital image acquisition technology. These preoperative planning software include EndoMap (Siemens AG, Medical Solutions, Erlangen, Germany) [5], IMPAX (Agfa Corporation, Mortsel, Belgium) [6], mediCAD (mediCAD Hectec GmbH, Landshut, Germany) [7, 8], TraumaCad (BrainLAB Feldkirchen, Germany) [9], ZedHip (Lexi Co., Tokyo, Japan) [10], Orthoview (Meridian Technique Ltd, Southampton, United Kingdom) [11], EOS (EOS™ Imaging, Paris, France) [12], and et al. These programs can be used to implement 2D and 3D preoperative planning.

Due to practical clinical constraints, X-rays are often preferred to develop a 2D preoperative planning for total hip arthroplasty. Using traditional template techniques is both time-consuming and less accurate, whereas using CT scans enhances accuracy to some degree. Guidelines for proper X-ray imaging are now available. For a standard anteroposterior pelvic view, the radiographic tube is directed cephalad at an angle of 10–15 degrees just above the pubic symphysis. The feet are internally rotated 15°. The symmetry of the femoral head, obturator foramen, ischial sphenoid, greater and lesser trochanter is noted on the image [13]. However, in clinical practice, the selection of prosthesis size is based on the position of the pelvis and femur, which may affect the correct implementation of the 2D preoperative planning.

This study used human phantoms to study the influence of pelvic and femoral factors on prosthesis size selection in preoperative planning of total hip arthroplasty. This study was divided into different positions of the pelvis and femur and into various angles of size, which was more systematic. This research aimed to determine the influence of pelvic and femoral positions on the selection of prosthesis size for preoperative planning of total hip arthroplasty and to provide a range of reference for clinical radiographs during preoperative planning of total hip arthroplasty.

## Materials and methods

### Radiometric setup

Classical radiographic imaging was used in this experiment, where standard AP radiographs were obtained through ceiling-fixed roentgen tubes with the parameters (90 kv, 4 mAs). Radiographs of each group would be taken in the same radiological parameter settings for each position. Additionally, the height of the X-ray tube and the table on which the experimental model was placed remained constant at 1 m. When filming, both lower limbs were naturally straight and shoulder-width apart (the midline of the body passes through the middle of the legs), both patellas were forward, and both lower limbs were internally rotated so that the toes of the feet were opposite each other (internal rotation of approximately 15°).

### Experimental model device

**Fig. 1:**
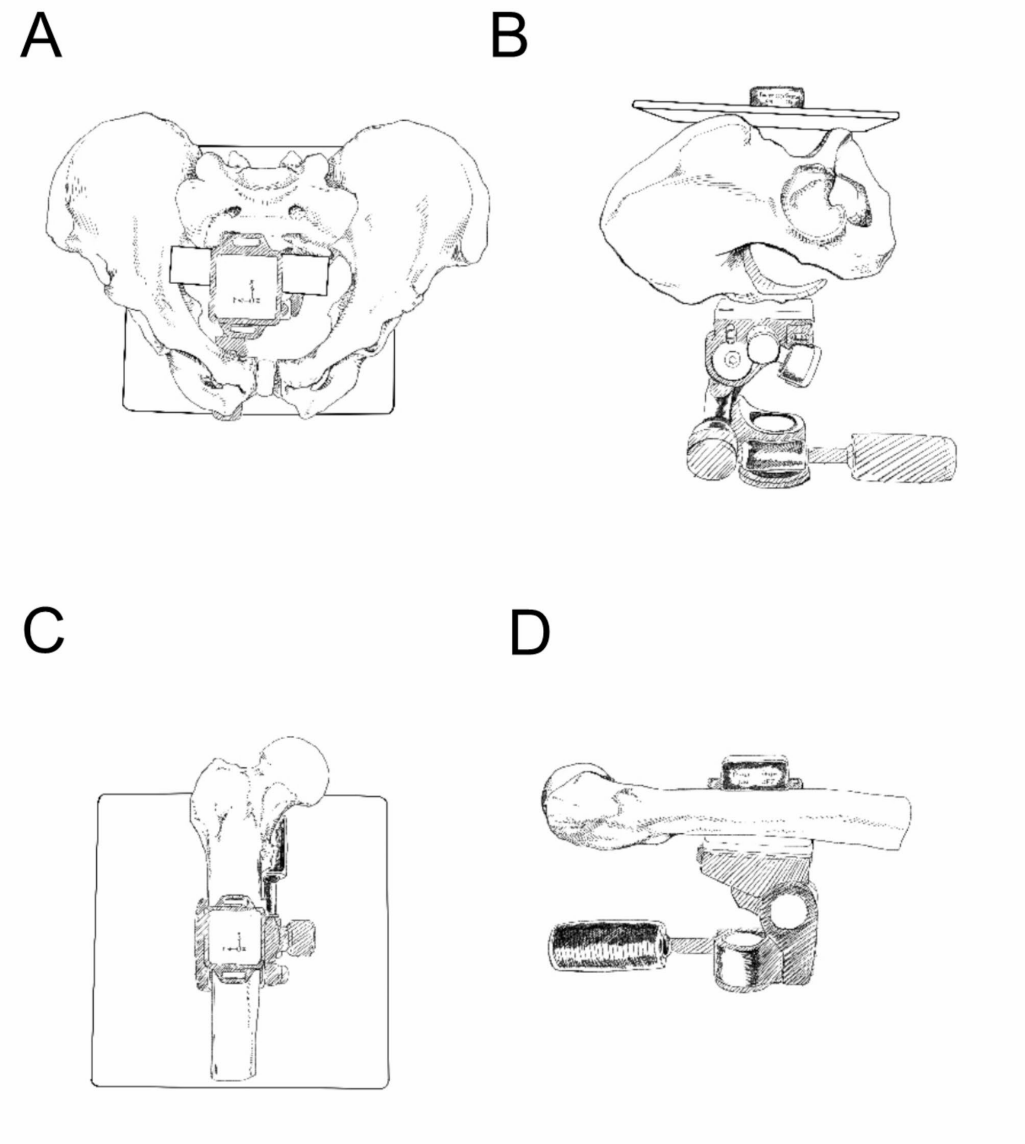
The pelvic device (**AB**) consists of a support base, a gimbal, a pelvic model, a Plexiglas plate, and a sensor. The femoral device (**CD**) consists of a support base, a gimbal, a femoral model and a sensor

This experimental setup consisted of a support base, a gimbal, a pelvis model, a femur model, a Plexiglas plate, and a sensor (Fig. 1). The model could control the pelvis to change in neutral, internal rotation, external rotation, left tilt, right tilt, anterior tilt, and posterior tilt positions, as well as the femur to change in neutral, internal rotation, external rotation, adduction, abduction, anterior flexion, and posterior extension positions. The sensor connected to a mobile phone via Bluetooth and accurately determined the angle of movement of the pelvis and femur in the X, Y and Z axes. Its accuracy was 0.2 degrees on XY and 0.5 degrees on the Z axis.

### Measuring protocol

To obtain a relatively uniform magnification of the radiographs, the distance between the x-ray radiographic bulb and the cassette was set at 1 m, and a steel ball with a diameter of 25 mm was used to place it below the pubic symphysis or near the greater trochanter of the femur. The pelvic and femoral parts of the experiment were performed separately. The pelvic and femoral models followed the same measuring protocol (Table 1). The exact angular values were precisely determined by the control gimbal in the experimental setup and by sensors glued to the pelvis or femur.

**Table 1:** Pelvic and femoral angle grouping protocol (unit: °)

Small angle group	large angle group
0.5	6
1.0	7
1.5	8
2.0	9
2.5	10
3.0	11
3.5	12
4.0	13
4.5	14
5.0	15

### Data analysis

**Fig. 2:**
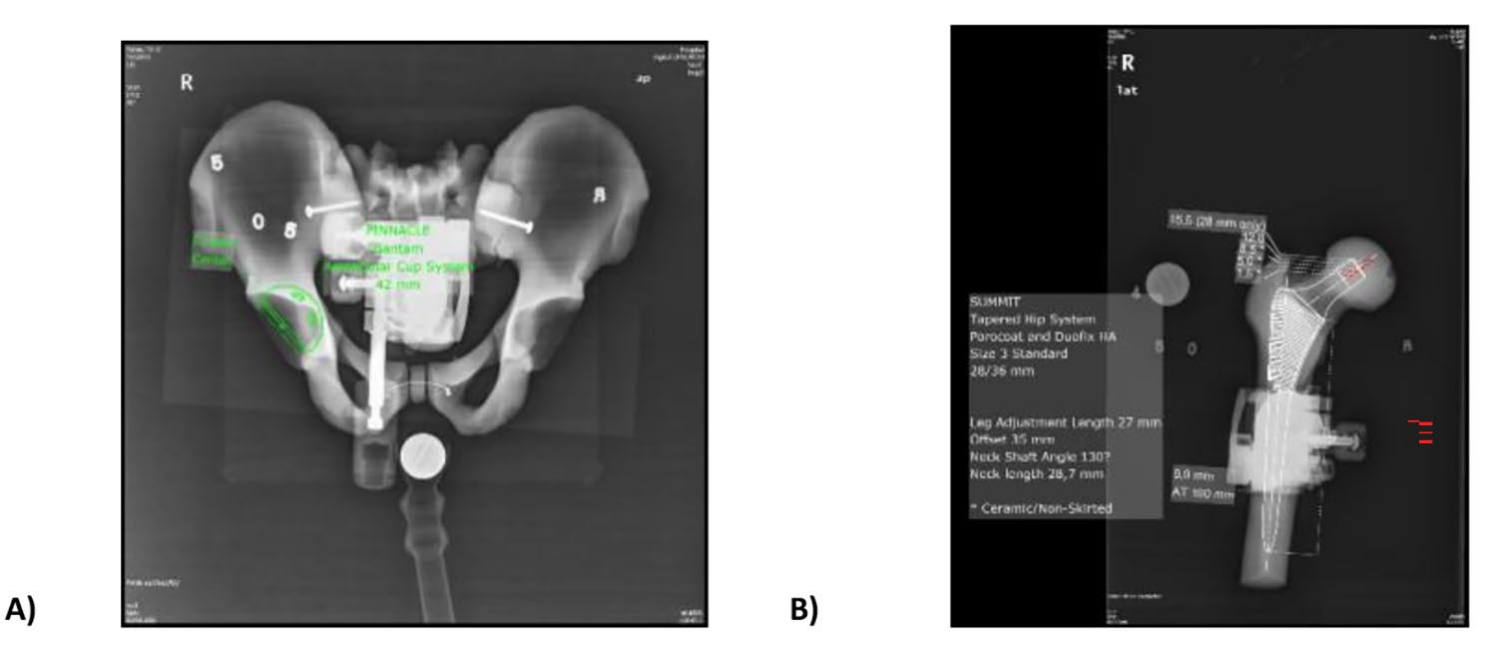
Fig. **A** displays the range of acetabular cup sizes available for measuring a left pelvic tilt of 0.5 degrees using the mediCAD software. Fig. **B** showcases the available options for femoral stem size when measuring a femoral posterior extension of 5.0 degrees using the mediCAD software

The diameter of the acetabular cup was measured using the anteroposterior pelvic view, while the size of the femoral stem prosthesis was determined using the anteroposterior femoral view. These measurements were obtained following the guidelines of the 2D program in the mediCAD software (mediCAD^®^ 2D hip module, v7.0, imediCAD(Beijing) Medical Technology Co., Ltd, China) (Fig. 2). Johnson & Johnson DePuy prostheses were utilised. We used a manual planning module to size the prosthesis following the method used to make the preoperative planning for total hip arthroplasty.

### Statistics

The statistics were analysed by SPSS (Version 23, SPSS Inc., Illinois, USA). The chi-square test was used to compare measurements on different pelvic or femoral positions, with a p-value less than 0.05 considered a statistically significant difference.

## Results

The position of the pelvis was categorised as neutral, left tilt and right tilt, anterior tilt and posterior tilt, and internal rotation and external rotation. With the pelvis in a neutral position, the acetabular cup measured 42 mm, while the cup sizes in other pelvic positions varied within a range of three sizes (Table 2). The position of the femur was categorised as neutral, abduction and adduction, anterior flexion and posterior extension, and internal rotation and external rotation. With the femur in a neutral position, the femoral stem measured the size 2, while the femoral stem sizes in other femoral positions varied within a range of two sizes (Table 3). Furthermore, the angles that cause changes in prosthesis size and the range of change sizes were listed (Table 4).

**Table 2:** Different pelvic positions and acetabular cup sizes (mm)

Neutral	Left tilt and right tilt	Anterior tilt and posterior tilt			Internal and external rotation			
42	42	42	44	46	42	44	46	48
	40 100%	6 15%	15 37.5%	19 47.5%	11 27.5%	13 32.5%	7 17.5%	9 22.5%

**Table 3:** Different femoral positions and femoral stem sizes

Neutral	Abduction and adduction	Anterior flexion and posterior extension		Internal and external rotation	
2	2	2	3	1	2
	40 100%	29 72.5%	11 27.5%	32 80%	8 20%

**Table 4:** Pelvic and femoral position and the angles that cause changes in prosthesis size

	Angles that cause changes in prosthesis size (degree)	Range of size variation
Left pelvic tilt	none	0
Right pelvic tilt	none	0
Anterior pelvic tilt	3.0	1 size
	9.0	2 sizes
Posterior pelvic tilt	1.0	1 size
	4.5	2 sizes
Internal pelvic rotation	6.0	1 size
External pelvic rotation	1.0	1 size
	2.5	2 sizes
	7	3 sizes
Femoral abduction	none	0
Femoral adduction	none	0
Anterior femoral flexion	none	0
Posterior femoral extension	5.0	1 size
Internal femoral rotation	2.0	-1 size
External femoral rotation	3.0	-1 size

**Fig. 3:**
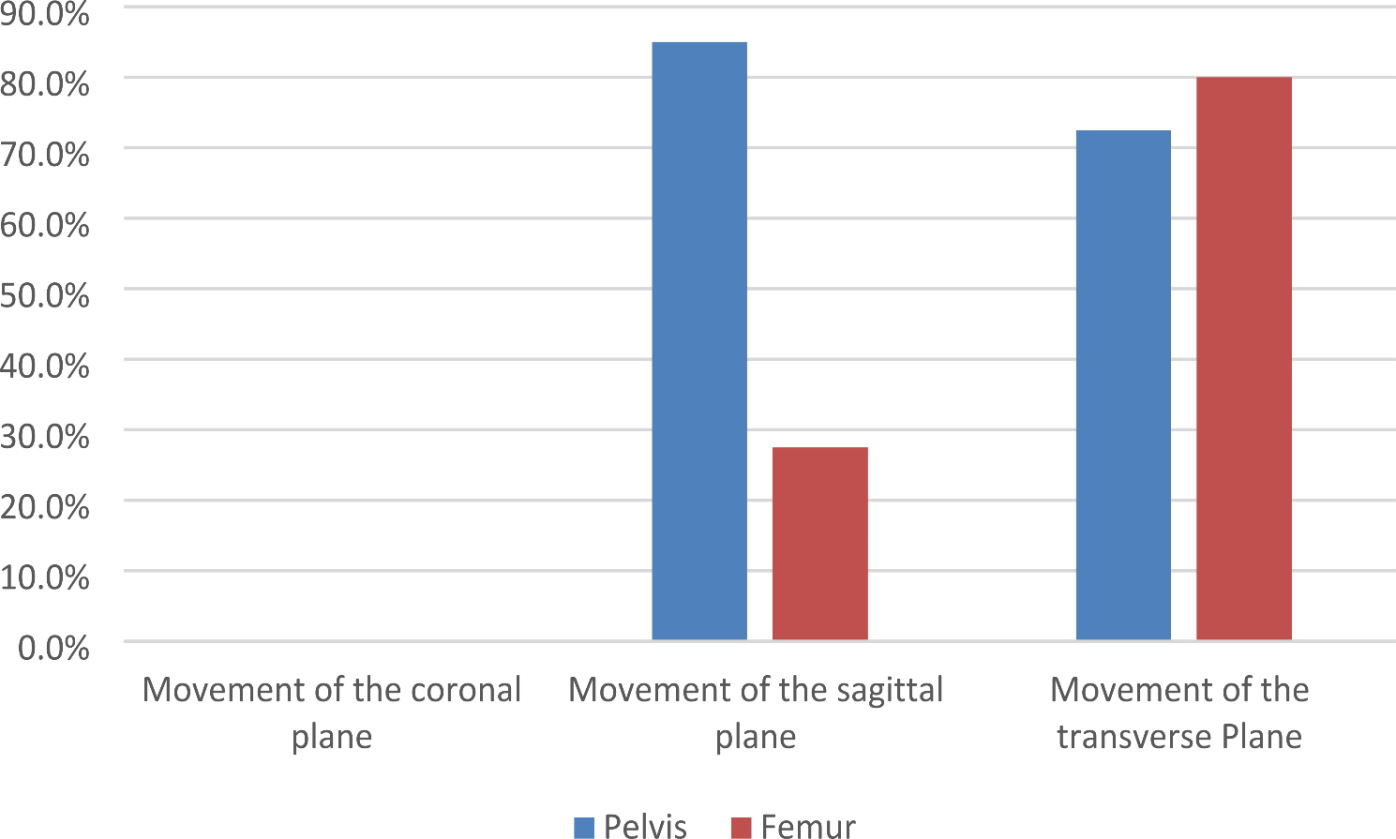
Box plot of the inconsistency rate between the pelvis and femur in different planes of prosthesis size selection relative to the neutral position (X-axis indicates movement in different planes, Y-axis indicates the inconsistency rate)

The left and right tilt of the pelvis and the abduction and adduction of the femur could be attributed to movements of the coronal plane. Anterior and posterior tilt of the pelvis and anterior flexion and posterior extension of the femur could be ascribed to movements of the sagittal plane. The internal and external rotation of the pelvis and femur could be regarded as movements of the transverse plane. The movement of the pelvis and femur in the coronal plane did not affect the choice of prosthesis size relative to the neutral position. Nevertheless, pelvic and femoral motions in the sagittal and transverse planes had a significant effect (Fig. 3 ).

The anteroposterior tilt of the pelvis compared to internal and external rotation was detected to be not significantly different using the chi-square test (*p* > 0.05). In contrast, the chi-square test revealed a significant difference in the anterior-posterior flexion and extension of the femur compared to internal and external rotation (*p* < 0.05).

## Discussion

Using a human phantom, this experiment investigated the position of the pelvis and femur on the choice of prosthesis size in preoperative planning. In the coronal plane, left-right pelvic tilt and femoral adduction and abduction did not affect the choice of prosthesis size. However, the selection of prosthesis size was influenced by the position of the pelvis and femur in both the sagittal and transverse planes. For the pelvis, the proportions of acetabular cup size inconsistencies between anterior and posterior pelvic tilt and internal and external pelvic rotation were: 85% and 72.5%, respectively. This high ratio suggests that the position of the pelvis highly influenced the size of the acetabular cup. For the femur, the proportion of femoral stem size inconsistencies between anterior flexion and posterior extension of the femur and internal and external rotation of the femur were: 27.5% and 80%, respectively. There was a statistically significant difference between these two groups, suggesting that the internal and external rotation of the femur was more susceptible.

A study examined 75 patients who underwent total hip arthroplasty and were followed up for one year postoperatively. The patients were categorised into three groups based on their preoperative pelvic tilt (anterior, intermediate, and posterior), and it was found that the presence of pelvic tilt during preoperative planning still yielded favourable THA results [14]. The finding of this study aligned to some extent with the results of the current experiment. Another study found that pelvic rotation of less than 20° was unlikely to have a clinical impact on preoperative measurements [15], but acetabular cup size was not included in preoperative measurements. Multiple studies have examined the relationship between pelvic tilt and acetabular cup position but have not addressed acetabular cup size [14, 16, 17].). Concerning the femur, a study showed a reference error of 7–8 mm for the greater trochanter at 20° of external rotation and flexion [18]. However, this study did not show an effect on the choice of femoral stem size during external rotation and flexion of the femur. Additionally, studies have indicated that errors caused by femoral adduction/abduction of less than 15° have limited clinical significance in measuring LLD [19]. However, the influence of femoral stem size on these errors has not been examined.

In this experiment, a 25 mm steel ball was used as a scale ball to solve the problem of X-ray magnification and ensure the accuracy of the experimental results. Meanwhile, this experiment demonstrated a more systematic approach by taking into account both pelvic and femoral alignment and categorising the magnitude angles. The number of patients with spondylopelvic pathology is growing [20], and when X-rays are taken, the pelvis and femur are often not in the standard neutral position. This project used a phantom to simulate the malalignment of the pelvis and femur in a clinical context. Furthermore, this experiment utilised a sensor attached to the pelvis and femur to precisely measure the angle of movement in both the pelvic and femoral regions.

This study focused on prosthesis size selection, and a review of the relevant literature revealed few articles that systematically investigated prosthesis size selection. It is widely recognised that an oversized acetabular cup may lead to bone destruction, looseness, and trigger pain. In contrast, an undersized acetabular cup may lead to joint instability, looseness, and possibly dislocation. Similarly, an oversized femoral stem may lead to femoral bone destruction, femoral fracture, nerve damage, or postoperative pain. In contrast, an undersized femoral stem may lead to loosening and sinking of the femoral stem, joint instability, or joint dislocation. While surgeons can make adjustments during surgery based on intraoperative conditions, it is essential to emphasise that thorough preoperative planning is crucial for ensuring the safety of the procedure.

Admittedly, using software from only one preoperative planning company and relying on one type of prosthesis in this study is a limitation. This experiment simulated the position of the pelvis and femur separately and did not comprehensively assess the pelvis and femur as a whole. In addition, just dividing into size-angle groups (from 0 to 15 degrees) did not match all scenarios in the clinic. Naturally, this experiment also lacked the assessment of intra-observer and inter-observer differences. However, the first author has already done research and published on the reproducibility and reliability of mediCAD software use at the doctoral stage [21]. Finally, it was worth noting that the acetabulum employed in this experiment was in a normal state and, as such, did not accurately replicate the presence of acetabular dysplasia. Our future work entails addressing the shortcomings above and broadening our research scope to encompass the knee and shoulder joints.

## Conclusions

Specific positions of the pelvis and femur can significantly influence the selection of the prosthesis size for preoperative planning. It is crucial for the radiographer to ensure that the pelvis and femur maintain a standard neutral position, particularly in the sagittal and transverse planes, during the image acquisition process.

## Data Availability

The data collected in this study will be stored in our hospital permanently. The data used or analysed during the current study is available from the corresponding author on reasonable request.
